# getDNB: identifying dynamic network biomarkers of hepatocellular carcinoma from time-varying gene regulations utilizing graph embedding techniques for anomaly detection

**DOI:** 10.1093/bioinformatics/btaf518

**Published:** 2025-09-15

**Authors:** Tong Wang, Zhi-Ping Liu

**Affiliations:** Department of Biomedical Engineering, School of Control Science and Engineering, Shandong University, Jinan, Shandong 250061, China; Department of Biomedical Engineering, School of Control Science and Engineering, Shandong University, Jinan, Shandong 250061, China

## Abstract

**Motivation:**

Early detection and timely intervention of hepatocellular carcinoma (HCC) are pivotal for improving patient prognosis. Current diagnostic approaches often detect HCC at later stages, thereby diminishing treatment efficacy. Recent advancements in high-throughput sequencing technology have vastly improved the identification of molecular markers via biological networks. However, existing methodologies frequently overlook the intricate gene interaction information in temporal gene regulatory networks. Therefore, our study proposes an algorithm model, getDNB, leveraging graph embedding technique (get) for anomaly detection in time-varying dynamic networks. The model aims to facilitate early HCC detection and propel precision medicine by recognizing dynamic network biomarker (DNB).

**Results:**

We proposed the getDNB model, which utilizes graph convolutional networks for graph embedding, mapping high-dimensional gene regulatory networks to low-dimensional feature vector spaces. By calculating gene anomaly degrees through an outlier score, and using the minimum dominant set algorithm alongside with the shortest path algorithm, we discovered DNBs and their associated networks in HCC. The getDNB model successfully pinpointed 33 HCC DNBs, effectively differentiating various temporal stages of HCC progression, and demonstrated robustness across numerous real HCC datasets. Functional enrichment analysis unveiled that these DNBs play critical roles in HCC occurrence and development, outperforming widely used feature selection algorithms.

**Availability and implementation:**

The source code and data can be found at https://github.com/zpliulab/getDNB.

## 1 Introduction

For a wide array of complex diseases, the developmental trajectory is prolonged and intricate, shaped by the interplay of genetic and environmental factors. For instance, hepatocellular carcinoma (HCC), the most prevalent type of liver cancer (Bray *et al.* 2024), typically evolves through a multi-step and multi-stage process, often encompassing stages such as chronic hepatitis associated with hepatitis B virus (HBV) infection, hepatocyte dysplasia, low-grade dysplastic nodules, high-grade dysplastic nodules, early HCC, and advanced HCC (Motola-Kuba *et al.* 2006). Currently, existing diagnostic approaches for HCC predominantly detect the disease at its advanced stages, leading to unfavorable prognoses for patients. However, with the relentless progression of high-throughput sequencing technologies and network analysis algorithms, the identification of biomarkers via network biology methods has become increasingly common (Liu 2016). It is evident that alterations in gene interactions during the progression of complex diseases can exert a profound influence on the organism (Liu and Wang 2024). Consequently, uncovering the key genes involved in the dynamic evolution of HCC is vital for early diagnosis, prompt intervention, and the practice of personalized medicine.

Analogous to the variations seen in nature biological processes, the emergence and progression of numerous complex diseases are neither linear nor constant; they involve specific mutations and critical thresholds. Consequently, the trajectory of disease development process can roughly be delineated into three states: normal, pre-disease, and disease (Liu *et al.* 2012). The pre-disease state represents a critical phase preceding the transition from normal to disease. When intervened upon and treated, this stage can be reversible, thus thwarting progression into more severe disease stages. Conversely, in the absence of intervention or the presence of factors that expedite disease progression, it may escalate into an irreversible disease state. Hence, identifying key biomarkers indicative of the pre-disease stage is paramount. This brings us to the concept of dynamic network biomarker (DNB) (Liu *et al.* 2012), whose identification methods harbor significant potential for early disease detection and prediction, especially for the early warning of complex diseases.

In recent years, methods rooted in the DNB theory have undergone extensively utilization and expansion, leading to numerous significant approaches. For example, Liu *et al.* (2012) incorporated the concept of information entropy (IE) into DNB analysis. By utilizing high-throughput data from multiple samples, they defined local network entropy based on state transitions, enabling the identification of critical shifts in disease progression and early warning signals of dominant subnetworks. Building on this, Lyu *et al.* (2024) proposed a new method of network information entropy edge (NIEE), which can detect key states or critical points of complex diseases by utilizing DNBs, sample-specific networks, and information entropy theory, and demonstrated DNBs as early warning signals in acute and chronic disease case studies. Despite the promising developments and outcomes of existing DNB-based methods for identifying early warnings and critical transitions in complex diseases, most fail to account for the high-dimensional complexity of biomolecular networks, thereby limiting the extraction of valuable information underlying networks and leading to neglect of their potential in DNB discovery and recognition.

To address the limitations of existing methods, this article introduced graph embedding technique to transform complex high-dimensional biomolecular networks into low-dimensional feature vector spaces. We subsequently performed clustering, computed outlier scores, and incorporated minimum dominating set and shortest path algorithms to identify HCC DNBs and their associated networks. Moreover, we defined a dynamic network index (DNI), which elucidate the rationale and validity of the identified HCC DNBs. The main contributions of our work are summarized below:

- We introduced a novel model, getDNB, for identifying HCC DNBs using graph embedding techniques for anomaly detection. This approach leverages GCN to utilize information about nodes and edges in time-varying dynamic gene regulatory networks. We assessed the abnormality of genes at each stage by calculating outlier scores using K-means clustering. The integration of minimum dominating set and the shortest path algorithms enhances the accuracy of the identified HCC DNBs and ensures the connectivity of the identified network.
- We proposed a definition for the DNI and introduce a temporal classification strategy. The DNI quantifies the information change of the identified DNBs within the temporal rewiring regulatory networks. The sequential stratification evaluates the classification effectiveness of the obtained DNBs in time-varying gene regulatory networks.
- The experimental dataset and the other two validation datasets are all HCC-related. We achieved superior performance compared with other methods on all three datasets, demonstrating the effectiveness and advantage of getDNB in identifying DNBs from temporal networks.

## 2 Materials and methods

### 2.1 Data

In our study, we integrated gene expression data from HCC patients, along with corresponding clinical data, from the Gene Expression Omnibus (GEO) (https://www.ncbi.nlm.nih.gov/geo/) and The Cancer Genome Atlas (TCGA) (https://cancergenome.nih.gov/). Table 1 summarizes the basic sample information for the experimental dataset (ID: GSE6764, comprising 75 patients) and the independent validation datasets (ID: GSE89377, with 107 patients; TCGA-LIHC, including 424 patients) used for HCC DNBs discovery.

**Table 1.** Statistics of basic information of the datasets used in this work.^a^

Datasets	Plat-forms	Total cases	Total genes	Number of stages	References
GSE6764	GPL570	75	37 999	8	Wurmbach *et al.* (2007)
GSE89377	GPL6947	107	48 803	10	Melé *et al.* (2015)
TCGA-LIHC	DCC	424	60 660	5	Weinstein *et al.* (2013)

a The total number of cases in the TCGA-LIHC dataset included the number of para-carcinoma tissue.

The experimental dataset, GSE6764, comprises microarray-based gene expression data from HCC tissues across eight pathological stages of HCV-induced HCC development: control, cirrhosis, low-grade dysplastic nodules, high-grade dysplastic nodules, very early HCC, early HCC, advanced HCC, and very advanced HCC. Based on the dataset analysis (Wurmbach *et al.* 2007), we excluded three liver cirrhosis samples from non-HCC patients to avoid potential confounding effects, as they showed no significant gene expression differences from the HCC-associated cirrhosis. A total of 72 patients were included in our analysis, as detailed in Table 1. The gene expression matrix was normalized using log2 transformation duplicate genes were removed, and a final set of 20 824 genes was retained.

Furthermore, we utilized GSE89377 and TCGA-LIHC gene expression data as validation datasets. The GSE89377 dataset is categorized into 10 stages based on HCC occurrence. To maintain data balance, we selected seven stages similar to those in the experimental dataset after preprocessing and hierarchical clustering of the samples. The same normalization and screening process for the gene expression matrix was applied to the validation data. For TCGA-LIHC, we classified the samples into up to five pathological stages based on the clinical data. Details of the datasets and their process are provided in Supplementary Information 1, available as supplementary data at *Bioinformatics* online and Table 1, available as supplementary data at *Bioinformatics* online.

### 2.2 Establish specific gene regulatory networks utilizing prior background regulations

To construct stage-specific gene regulatory networks, we identified 2676 differentially expressed genes across eight pathological stages using ANOVA (Kim 2017) with an adjusted *P*-value threshold of <.0001. Given the importance of prior knowledge for biomarker selection, we integrated 170 relevant genes from the KEGG (Kanehisa *et al.* 2017) pathway for HCC into the former gene set. Furthermore, we utilized the RegNetwork (Liu *et al.* 2015) as a prior gene network background, which integrates the human gene regulatory networks from >25 databases. By mapping the collected HCC gene set onto the background network, we obtained an original network consisting of 2084 genes and 7534 interactions, which serves as the basis for generating gene regulatory networks for temporal stages respectively.

Using the gene expression matrix and the original network, we applied the PC-CMI algorithm (Zhang *et al.* 2012) to eliminate false positive edges, resulting in specific regulatory networks for each temporal stage respectively. Subsequently, we calculated the Pearson’s correlation coefficient between connected gene nodes as the edge weight, yielding weighted gene networks for each pathological stage. These time-varying networks reflected gene cooperation rewiring across HCC progression.

### 2.3 Framework

**Figure 1.**
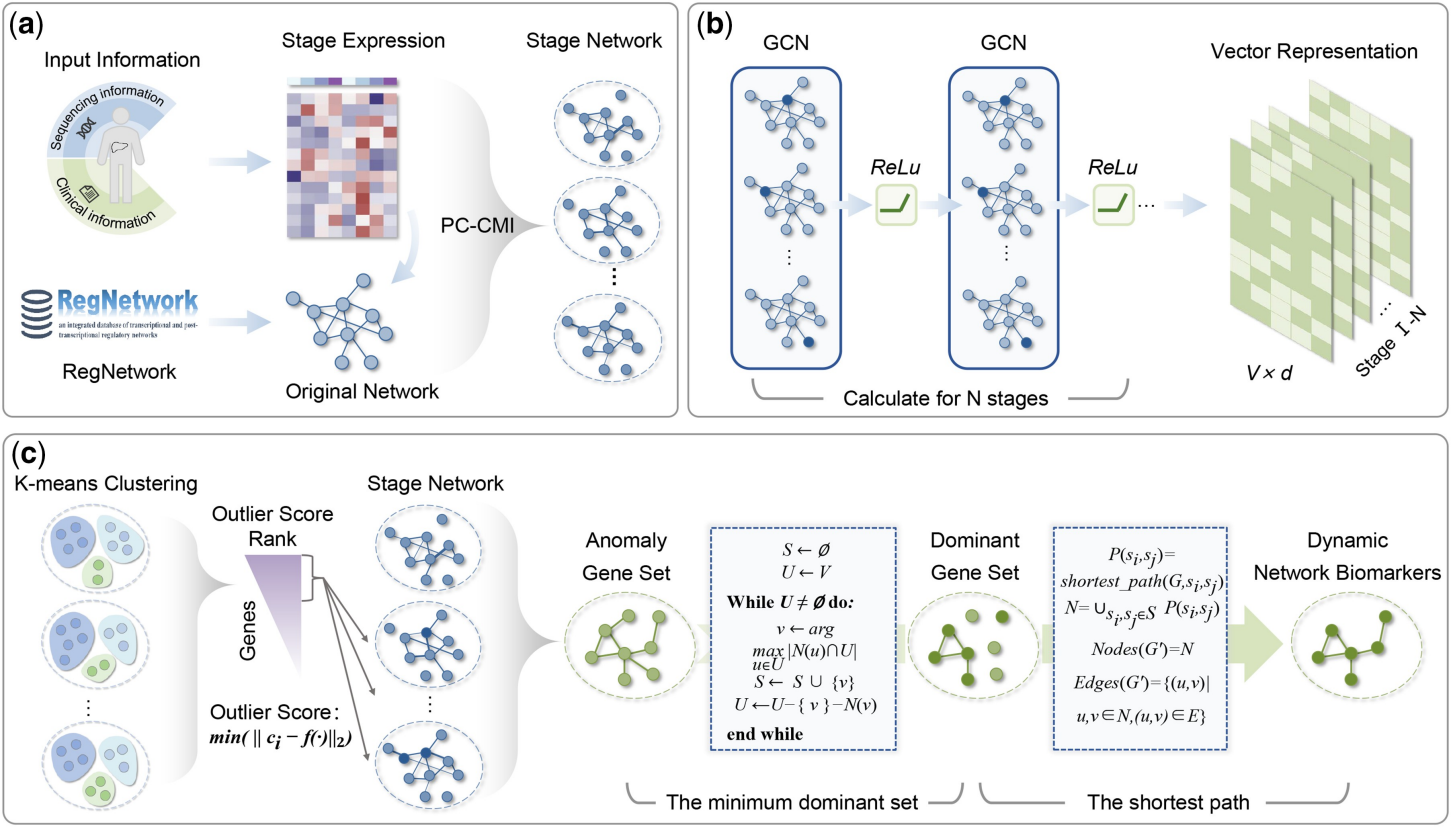
Framework of identifying HCC DNBs from temporal gene regulatory networks using getDNB. (a) Construction of specific gene regulatory networks across various pathological stages using the PC-CMI algorithm, based on integrated and preprocessed input data. (b) Graph embedding with GCN to transform high-dimensional gene networks into low-dimensional vector representations. (c) Identification of anomaly genes via K-means clustering and outlier score calculation, followed by refinement using the minimum dominant set and shortest path algorithms to obtain the final HCC DNBs and their interconnected regulations.

Figure 1 delineates an overview of getDNB for identifying HCC DNBs from gene regulatory networks and RNA-seq data. The framework encompasses three primary components: Firstly, an original network is constructed by the integration and preprocessing of input data. Leveraging the original network and gene expression matrix, the PC-CMI algorithm is implemented to derive specific gene interaction networks corresponding to pathological stages individually (Fig. 1a). Subsequently, these networks serve as input for the getDNB model, wherein GCN is used for graph embedding. This process transforms high-dimensional information from intricate gene regulatory interactions into low-dimensional vector space (Fig. 1b). Following this, K-means clustering is applied to the low-dimensional vectors of these dynamic time-varying networks to ascertain clustering information for each stage. Based on clustering, outlier scores for all genes (excluding the first stage) are computed. The getDNB model then screens anomaly genes in each stage based on their outlier scores, integrating them into an anomaly gene set. Given that the anomaly gene set might be extensive and contain disconnected nodes, the minimum dominant set algorithm and the shortest path algorithm are invoked to pinpoint the crucial genes (Fig. 1c). Ultimately, joint with these corresponding interconnected linkages, they serve as the discovered HCC DNBs.

### 2.4 Details of getDNB

#### 2.4.1 Graph embedding

The high dimensionality and structural complexity of biomolecular networks pose significant challenges to the direct discovery of DNBs. To address these challenges, graph embedding techniques have emerged as a promising solution (Cai *et al.* 2018). Graph embedding techniques map node information into a low-dimensional vector space while preserving the essential attributes of these nodes. Initially, these methods primarily relied on matrix decomposition (Cao *et al.* 2015) and random walk algorithms (Perozzi *et al.* 2014) to generate low-dimensional vector representations. However, these approaches often concentrated solely on the graph structure, neglecting other information and properties within the graph.

In recent years, neural network models have achieved remarkable success across diverse fields, prompting the introduction of neural network architectures into the domain of graph embedding (Kipf 2016, Kipf and Welling 2016, Veličković *et al.* 2017). The application of neural networks in graph embedding leverages the robust capabilities of deep learning to transform complex graph information underlying nodes, edges and their interactions into low-dimensional representation vectors. This transformation facilitates a broad spectrum of downstreamtasks. Notably, the GCN stands out as an effective graph embedding method (Kipf 2016). By applying convolution operations on graphs, GCN learns node representations by aggregating the features of neighboring nodes into the current node. This enables GCN to capture both structural and content features of the graph. Consequently, the proposed getDNB model utilizes GCN for graph embedding, as described below.

Given a graph, where G=(V,E) represents the set of nodes and *E* represents the set of edges. The nodes can be expressed as $V=\{v_{1},v_{2},\ldots ,v_{n}\}$, where N=|V| is the number of nodes. The edge set can be expressed as $E=\{v_{i},v_{j},w_{i,j}|v_{i},v_{j}\in V\}$, where $v_{i}$ and $v_{j}$ are two nodes, and $w_{i,j}$ represents the connection weight between the two nodes.

First, it maps the input nodes to integer indices and initialize the embedding vector of each node as a *d*-dimensional vector 


(1) $$H^{\left(0\right)}=E\in R^{\left(N\times d\right)},$$

where *d* represents the embedding dimension, *E* is the embedding matrix of size N×d, with each row corresponding to the initial embedding vector of a node.

Next, the graph convolution operation is applied. For the input embedding $H^{\left(l\right)}$ at the *l*th layer, the output of the graph convolution is


(2) $$H^{\left(l+1\right)}=\sigma (AH^{\left(l\right)}W^{\left(l\right)}),$$

where $A={\tilde{D}}^{-\frac{1}{2}}\tilde{A}{\tilde{D}}^{-\frac{1}{2}}$ is the normalized adjacency matrix. $\tilde{A}$ is *A* with self-loops included, $\tilde{D}$ is the pair Angle matrix, and is $W^{\left(l\right)}$ the weight matrix of the *l*th layer, and σ is the activation function, specifically the Rectified Linear Unit (ReLU) (Nair and Hinton 2010).

The loss between the model output and the target embedding is computed by using the mean square error (MSE) loss function, i.e.


(3) $$l=\frac{1}{N}\sum_{i=1}^{N}\left|\right|H_{i}^{\left(L\right)}-E_{i}|{|}^{2},$$

where *L* is the last layer of the model.

Based on the calculated loss, we used the Adam optimizer (Mathieu *et al.* 2015) to minimize the loss function:


(4) $$\theta^{\left(t+1\right)}=\theta^{\left(t\right)}-\eta \nabla_{\theta }l,$$

where the θ is the parameter of the model, and η is the learning rate, and $\nabla_{\theta }l$ is the gradient of the loss function with respect to the model parameters.

Repeat the above process several times until the loss converges. The node embeddings of the last layer of the output GCN serve as the final obtained low-dimensional vector representation, which can be used for down-stream tasks.

#### 2.4.2 Anomaly detection for biomarkers

The network representation obtained by getDNB through graph embedding using GCNs serves as the foundation for anomaly detection. In our model, we defined the outlier score of nodes in a dynamic gene regulatory network as the minimum Euclidean distance from a node to the center of its nearest cluster, specifically within the context of HCC disease progression. The specific implementation is as follows.

Given a biomolecular network at a temporal stage *t*, the low-dimensional vector representation of each node $v_{i}$ is denoted as $h_{i}^{\left(t\right)}$. Based on these vector representations, we performed K-means clustering (McQueen 1967) to divide all nodes into k clusters, denoted as $\left\{C_{1}^{\left(t\right)},C_{2}^{\left(t\right)},\ldots ,C_{k}^{\left(t\right)}\right\}$. For each cluster $C_{j}^{\left(t\right)}$, the center vector $c_{j}^{\left(t\right)}$ is calculated using the formula:


(5) $$c_{j}^{\left(t\right)}=\frac{1}{\left|C_{j}^{\left(t\right)}\right|}\sum_{v_{i}\in C_{j}^{\left(t\right)}}h_{i}^{\left(t\right)},$$

where $\left|C_{j}^{\left(t\right)}\right|$ represents the number of nodes in cluster $C_{j}^{\left(t\right)}$. For each cluster $C_{j}^{\left(t\right)}$, the node $v_{c_{j}}^{\left(t\right)}$ closest to the cluster center is selected as the representative cluster center node by minimizing the distance to the center:


(6) $$v_{c_{j}}^{\left(t\right)}=\arg\underset{v_{i}\in C_{j}^{\left(t\right)}}{\min}\left\|h_{i}^{\left(t\right)}-c_{j}^{\left(t\right)}\right\|.$$

Next, at temporal stage t+1, where the lower-dimensional vector of each node $v_{i}$ is denoted as $h_{i}^{\left(t\right)}$, we computed the Euclidean distance between node $v_{i}$ and the center of each cluster specified at stage *t*, and then took the smallest of these distances as the outlier score for the node $v_{i}$ at stage t+1, i.e.


(7) $$\mathrm{Outlier}\;\mathrm{Score}(v_{i},t+1)=\underset{j\in k}{\min}\left\|h_{i}^{\left(t+1\right)}-h_{c_{j}^{t}}^{\left(t+1\right)}\right\|,\;t\geq 0.$$

By analogy, we can calculate the outlier scores for nodes in the network for all temporal stages except the first stage and select anomaly genes at each subsequent stage can be completed based on a predefined proportion with the highest outlier scores.

#### 2.4.3 Crucial point extraction

Selecting anomaly genes based on a fixed proportion may introduce redundancy, and the resulting anomaly gene set may not form a fully connected network. Therefore, the extraction of crucial points in the getDNB model is of great importance.

To mitigate redundancy within the anomaly gene set, we utilized the Depth-First Search (DFS) greedy algorithm (Tarjan 1972) to extract the minimum dominating set from the anomaly candidates. Since the minimum dominating set may not necessarily form a connected network, the Dijkstra algorithm (Dijkstra 2022) was used to find the shortest paths in getDNB. After computing, we completed the crucial point extraction process and obtain the discovered HCC DNBs along with their connected network. The specific algorithm process and pseudo code are outlined in Supplementary Information 2, available as supplementary data at *Bioinformatics* online.

### 2.5 Performance evaluation

#### 2.5.1 Dynamic network index

Utilizing the outlier score, we can easily assess the abnormality level of each gene across temporal stage and pinpoint HCC DNBs through crucial point extraction methods. However, it is important to quantify the efficacy of the identified DNBs within the dynamic network. According to the definition of DNBs, in the pre-disease stage, the expression of DNBs is anticipated to be disrupted and chaotic (Liu *et al.* 2012). Given that entropy serves as a metric for disorder, disorganization, or uncertainty, it naturally suggests itself as a measure for evaluating the effectiveness of our selected biomarkers for HCC. Herein, we adapted and expanded the concept of community entropy (Yang *et al.* 2024) to introduce a novel dynamic network index (DNI):


(8) $$DNI_{i}=\text{exp}(–CE_{i}),$$

where $CE_{i}$ denotes the community entropy within a biomolecular network, defined as:


(9) $$CE_{i}=–\frac{g_{\alpha_{i}}}{D\left(G_{o}\right)}{\;\text{log}\;}_{2}\frac{D\left(\alpha_{i}\right)}{D\left(G_{o}\right)}.$$

Here, *i* refers to a specific temporal stage, $G_{o}$ denotes the amalgamation of all temporal stage networks, $\alpha_{i}$ refers to a module within the temporal *i*, and $g_{\alpha_{i}}$ denotes to the summation of the absolute PCC values of all nodes within the module and their first-order neighbors, excluding nodes that belong to the module itself. Alternatively, it can be interpreted as the summation of the absolute weight values of all edges, excluding the internal edges of the module. The function *D* calculates the total sum of node degrees across the graph.

Community entropy quantifies the cohesiveness within modules. Modules with stronger internal connections and weaker interactions with external nodes exhibit higher cohesiveness, resulting in a larger DNI. An increase in DNI signifies an augmentation in the degree of disorder within biomolecular networks. The observation of a tipping point or mutation in the DNI suggests a high likelihood that the corresponding temporal stage marks the pre-disease stage we are seeking for.

#### 2.5.2 Temporal classification

In the identification of HCC DNBs, the gene expression data utilized can be viewed temporal stage data from a broader perspective. Unlike conventional multi-type data, temporal stage data processes unique characteristics such as inherent order, time dependency, and diversity, which may render traditional classification algorithms less effective in processing it. Consequently, we introduced a classification strategy tailored for temporal stage data, leveraging the support vector machine (SVM) (Hearst *et al.* 1998) to evaluate the classification performance of features in these data.

The method is outlined as follows: when classifying temporal stage data, the dataset is split into two parts based on time, and the average result of these parts is adopted as the ultimate classification output. Specifically, for a given series of temporal stages, the data from the 1 to N−1 stage is labeled as 1, while the data from N−1 to *N* is labeled as 0. Upon completion of all N−1 binary classifications, the mean and standard deviation of the classification results are computed to determine the final classification outcome.

Addressing the challenge of data imbalance in the application of the temporal classification, we incorporated additional classification evaluation metrics beyond the AUROC (Area Under the Receiver Operating Characteristic Curve). To provide a more comprehensive assessment of the classification performance of the selected features, we also considered AUPRC (Area Under the Precision-Recall Curve), Accuracy, F1-score, Precision, and Recall as evaluation metrics.

#### 2.5.3 Functional enrichment analysis

To confirm the functionalities of these selected HCC DNBs, we conducted gene ontology (GO) (Gene Ontology Consortium 2021) enrichment analysis and KEGG (Kanehisa *et al.* 2017) pathway enrichment analysis. Furthermore, we used Metascape (http://metascape.org/) to contrast the functional enrichment results for HCC DNBs with those identified by other methods.

## 3 Results and discussion

### 3.1 Selected HCC DNBs and the interconnected networks

We utilized the time-varying networks, derived from the original gene regulatory network, as input for the GCN component of getDNB for graph embedding. The input data was split into a training set and a test set with an 8:2 ratio, and the model underwent training for 100 epochs until the loss function converged. The network representation obtained through graph embedding serves for K-means clustering. Prior to clustering, we determined the optimal number of clusters to be 24 using the elbow method. Consequently, we identified the 24 cluster centers during the clustering process. Based on the node vectors of the cluster center and the current time point, we computed the outlier scores for the nodes across the 7 temporal stages, excluding the initial stage. Given the dataset’s size, we selected the union of genes in the top 1% of outlier scores across these 7 temporal stages as the anomaly gene set. This gene set was then mapped onto the network union across all temporal stages, yielding a network consisting of 53 nodes and 172 edges. After applying the minimum dominant set and the shortest path algorithms, we ultimately identified 33 genes as HCC DNBs, accompanied by a connected network comprising 122 edges.

**Figure 2.**
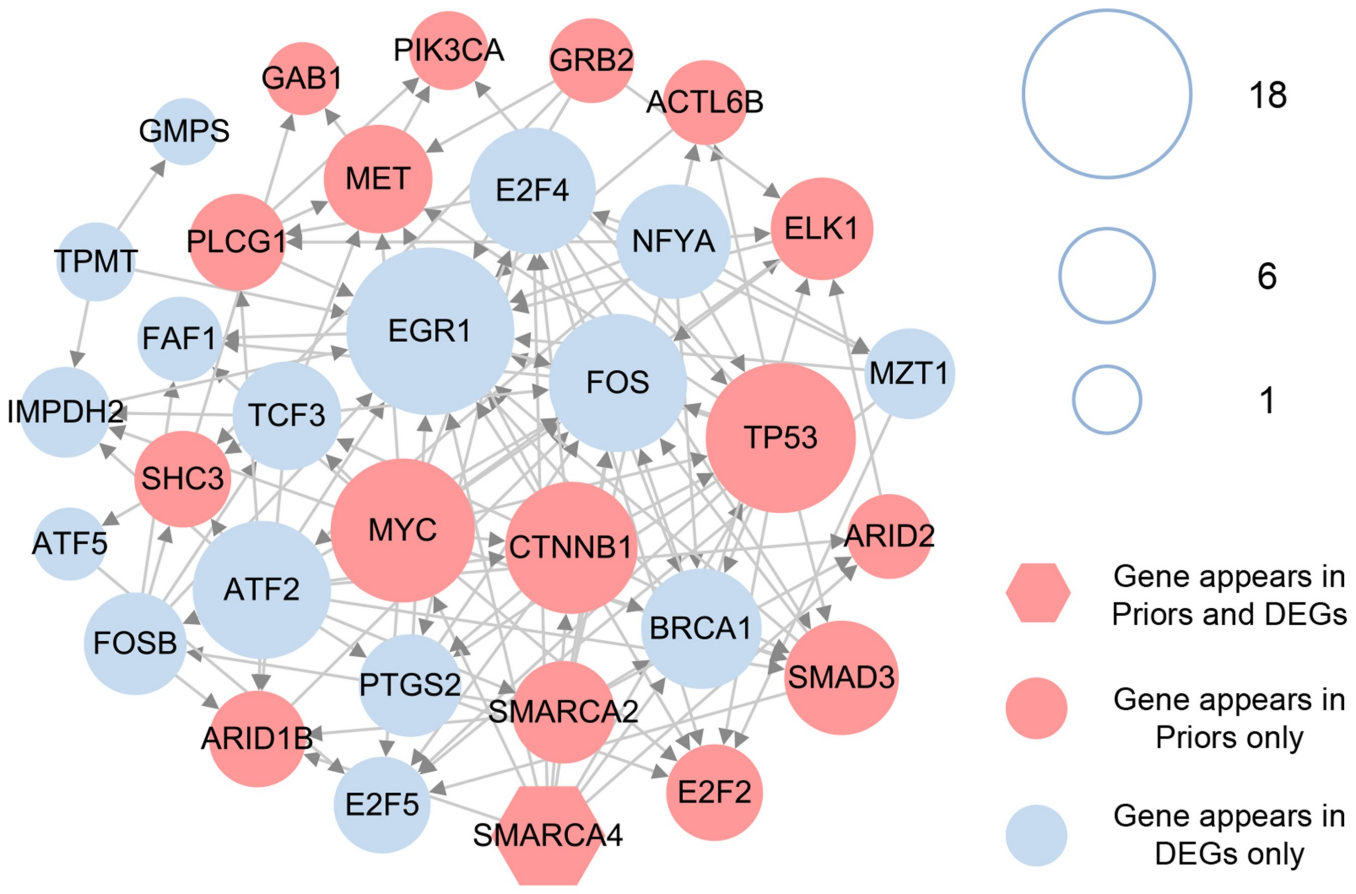
Network structure of 33 HCC DNBs. The color and shape of nodes represent gene attributes, while node size indicates the degree of interaction.

We visualized the connected network of these selected HCC DNBs, showing the gene degree within the network and their acquisition source (as depicted in Fig. 2). The size of the nodes reflects the degree of each gene. Among the 33 HCC DNBs, 16 genes were exclusively identified as differentially expressed genes, 16 genes were exclusively found in the prior HCC disease genes, and 1 gene belonged to both categories. Furthermore, we explored the correlation between these genes and HCC in existing literature and discovered that several of them have been previously confirmed to be associated with the occurrence and progression of HCC (refer to Table 2, available as supplementary data at *Bioinformatics* online for details).

**Table 2.** Temporal classification performance of five feature selection methods and two DNB identification methods on GSE89377 dataset.

Methods	AUROC	AUPRC	Accuracy	F1-score	Precision	Recall
getDNB	**0.9293 ± 0.0019**	**0.9579 ± 0.0009**	0.8409 ± 0.0040	**0.8468 ± 0.0037**	**0.8616 ± 0.0084**	0.8404 ± 0.0057
IE-DNB	0.8511 ± 0.0057	0.8807 ± 0.0084	0.7879 ± 0.0073	0.7704 ± 0.0175	0.7345 ± 0.0222	0.8236 ± 0.0169
NIEE-DNB	0.8884 ± 0.0046	0.8365 ± 0.0247	0.8561 ± 0.0051	0.7809 ± 0.0180	0.7913 ± 0.0414	0.8090 ± 0.0203
DEG(benchmark)	0.8831 ± 0.0071	0.9212 ± 0.0048	0.8106 ± 0.0127	0.7894 ± 0.0226	0.8566 ± 0.0173	0.7413 ± 0.0310
DUBStepR	0.8695 ± 0.0096	0.8821 ± 0.0071	0.8182 ± 0.0028	0.7837 ± 0.0194	0.8181 ± 0.0215	0.7756 ± 0.0340
RF-RFE	0.8387 ± 0.0094	0.9157 ± 0.0028	0.7879 ± 0.0122	0.8172 ± 0.0069	0.7844 ± 0.0204	**0.8766 ± 0.0089**
sPLS-DA	0.9173 ± 0.0038	0.9237 ± 0.0073	**0.8561 ± 0.0024**	0.8436 ± 0.0062	0.8574 ± 0.0147	0.8463 ± 0.0116
SVM-RFE	0.8858 ± 0.0084	0.8642 ± 0.0272	0.8022 ± 0.0060	0.8343 ± 0.0206	0.8343 ± 0.0206	0.7753 ± 0.0211

Values in bold represent the maximum value.

### 3.2 Classification results

**Figure 3.**
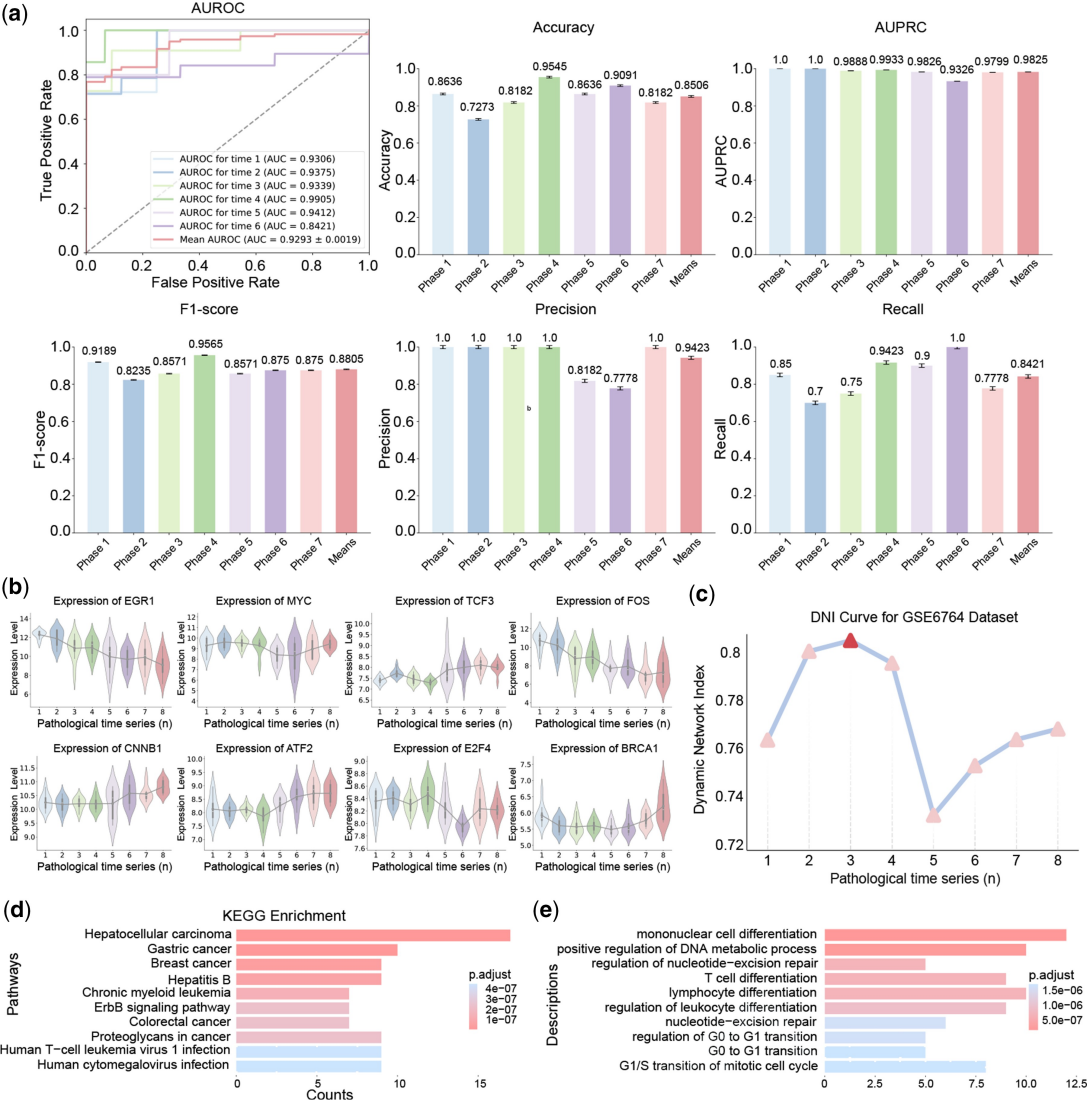
Comprehensive evaluation of HCC DNBs in the GES6764 dataset. (a) AUROC curve demonstrating the effectiveness of HCC DNBs selected by getDNB in distinguishing different pathological stages of HCC. (b) Violin plots showing expression levels of the top eight HCC DNB genes across temporal stages. (c) Temporal DNI curve, with a significant peak at the third stage, suggesting it as the critical point of transiting from pre-disease stage and disease stage. (d) KEGG pathway and (e) GO enrichment analyses of the 33 selected genes, highlighting the top 10 functions by adjusted *P*-values.

To validate the effectiveness of the selected HCC DNBs in distinguishing the occurrence and development of HCC, we used the temporal classification method outlined in the preceding section to compute the classification indices (shown in Fig. 3a). The results indicated that the HCC DNBs selected by getDNB exhibited robust performance in differentiating various pathological stages, confirming that these DNBs can effectively discern the distinct stages of HCC development.

Furthermore, to visually illustrate the variations in expression levels of the selected HCC DNBs across temporal stages, we generated violin plots that showcase the expression of the top eight genes, ranked by nodal degree within the HCC DNBs, across the temporal stages (displayed in Fig. 3b).

### 3.3 The temporal DNI curve

To assess the efficacy of the HCC DNBs identified by getDNB, we utilized the proposed DNI metric and constructed the temporal DNI curve for the HCC DNBs (illustrated in Fig. 3c). Notably, the temporal DNI curve exhibits a prominent peak during the third temporal stage, highlighting a significant transition. This observation indicates that the third stage is potentially the pre-disease stage in the progression of HCC. Upon deeper analysis, we discovered that the third stage aligns with the stage of low-grade dysplastic nodules. During this stage, most nodules either regress to normalcy or evolve into high-grade dysplastic nodules. While highly atypical nodules tend to be monoclonal and frequently advance to early HCC. Importantly, with prompt and suitable intervention, many of these nodules have the potential to revert to normal.

Thus, we preliminarily conclude that the HCC DNBs identified by the proposed getDNB model effectively categorize the pre-disease stage within dynamic networks and provide meaningful interpretability.

### 3.4 Functional enrichment analysis

To gain deeper insights into the functional relevance of the selected HCC DNBs, we conducted comprehensive KEGG pathway enrichment analysis and GO enrichment analysis on the 33 identified genes. The top 10 functions were displayed based on the adjusted *P*-values (depicted in Fig. 3d and e).

In the KEGG pathway enrichment analysis, the most significantly enriched pathway was hepatocellular carcinoma, solidifying the robust correlation between the HCC DNBs and the disease. Additionally, pathways such as Hepatitis B (Bréchot *et al.* 2000), ErbB signaling pathway (Berasain *et al.* 2007), and Proteoglycans in cancer (Baghy *et al.* 2016) were also enriched, all of which are intimately associated with the progression of HCC.

The GO enrichment analysis further revealed biological processes that are pertinent to HCC. Notably, Positive regulation of DNA metabolic process (Yang *et al.* 2020) emerged, where disruptions in DNA repair, replication, and recombination processes can expedite HCC development. Regulation of nucleotide-excision repair (Fautrel *et al.* 2005), when impaired, can lead to the accumulation of DNA damage, ultimately culminating in cancer, including HCC. Similarly, the G1/S transition of mitotic cell cycle (Greenbaum 2004) is frequently dysregulated in HCC, with mutations or abnormal expression of cell cycle regulatory proteins during this phase fostering tumor cell proliferation. These GO terms are pivotal processes in progression of HCC.

### 3.5 Independent validations

To ascertain the validity and reliability the HCC DNBs identified by our proposed method, we used the TCGA-LIHC dataset and the GSE89377 HCC dataset for validation.

**Figure 4.**
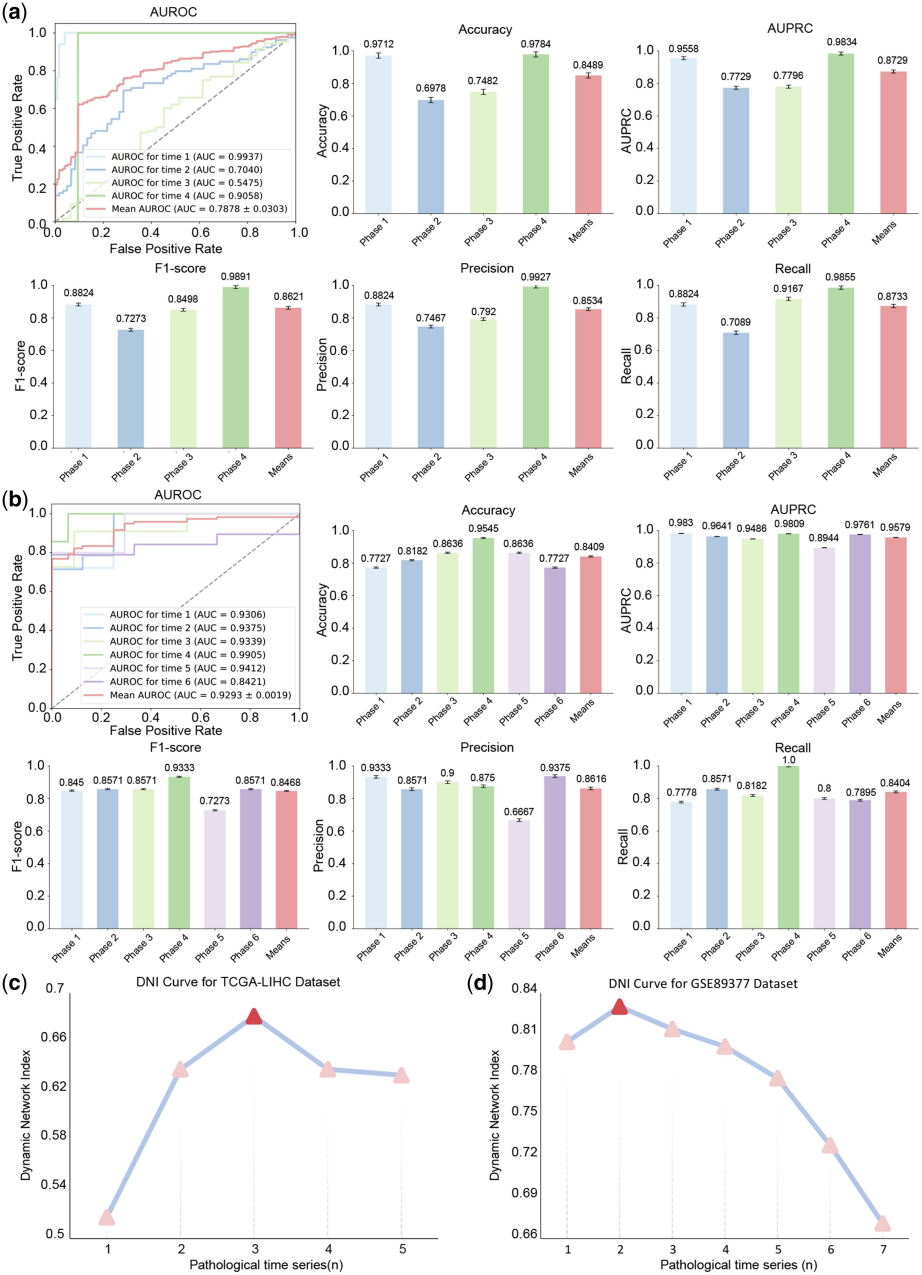
Comprehensive evaluation of HCC DNBs in multiple datasets. (a) Temporal classification performance of HCC DNBs in the TCGA-LIHC dataset. (b) Temporal classification performance of HCC DNBs in the GSE89377 dataset. (c) DNI curve of HCC DNBs in the TCGA-LIHC dataset, highlighting transition points. (d) DNI curve of HCC DNBs in the GSE89377 dataset, also indicating transition points.

Initially, we assessed the temporal classification performance of the HCC DNBs on these two datasets (refer to Fig. 4a and b). The results clearly demonstrated that the HCC DNBs exhibited robust classification performance across the independent datasets, affirming the resilience of the HCC DNBs identified by getDNB and their applicability to new samples.

Furthermore, we plotted the DNI curves for the HCC DNBs for both the TCGA-LIHC and GSE89377 datasets. As illustrated in Fig. 4c and d, the HCC DNBs selected by getDNB displayed transition points in both datasets, occurring at early pathological stages with considerable interpretability. This indicated that the HCC DNBs identified by getDNB can proficiently delineate the pre-disease stage within dynamic change networks across diverse real-world datasets.

### 3.6 Compared with other methods

To evaluate the advancements and accuracy of our model, we conducted a comparative analysis with several widely used feature selection methods, namely DUBStepR (Ranjan *et al.* 2021), RF-RFE (Breiman 2001), DEG (benchmark) (Love *et al.* 2014), sPLS-DA (Lê *et al.* 2011), SVM-RFE (Sanz *et al.* 2018), and the existing DNB identification methods, such as IE-DNB (Liu *et al.* 2012) and NIEE-DNB (Lyu *et al.* 2024). We applied these methods to select gene sets in the experimental dataset and then performed subsequent analysis on the independent validation datasets. Details of the procedures are provided in Supplementary Information 3, available as supplementary data at *Bioinformatics* online.

First, we compared the overlapping genes in the gene sets selected by different feature selection methods and analyzed the KEGG and GO functional enrichment of the gene sets selected by these methods (refer to Fig. 2, available as supplementary data at *Bioinformatics* online). It is evident that getDNB demonstrated significant enrichment in HCC pathways, whereas the gene sets selected by other methods did not show favorable enrichment results associated with HCC.

More importantly, we compared the temporal classification performance of the biomarkers selected by these six methods on two independent datasets (see Tables 2 and 3). The information presented in the tables reveals that, for both independent validation datasets, the evaluation metrics for HCC DNBs selected by getDNB are generally superior to other methods.

**Table 3.** Temporal classification performance of five feature selection methods and two DNB identification methods on TCGA-LIHC dataset.

Methods	AUROC	AUPRC	Accuracy	F1-score	Precision	Recall
getDNB	**0.7878 ± 0.0303**	**0.8729 ± 0.0094**	**0.8489 ± 0.0162**	**0.8621 ± 0.0087**	0.8634 ± 0.0089	0.8733 ± 0.0104
IE-DNB	0.7124 ± 0.0233	0.8236 ± 0.0111	0.8219 ± 0.0212	0.8305 ± 0.0125	0.7796 ± 0.0177	**0.8955 ± 0.0118**
NIEE-DNB	0.7680 ± 0.0198	0.8492 ± 0.0074	0.8273 ± 0.0208	0.8356 ± 0.0122	0.8158 ± 0.0137	0.8598 ± 0.0138
DEG(benchmark)	0.7603 ± 0.0228	0.8294 ± 0.0095	0.8327 ± 0.0195	0.8442 ± 0.0101	0.8384 ± 0.0113	0.8532 ± 0.0115
DUBStepR	0.7686 ± 0.0260	0.8598 ± 0.0084	0.8219 ± 0.0240	0.8234 ± 0.0145	0.8038 ± 0.0137	0.8464 ± 0.0173
RF-RFE	0.7141 ± 0.0276	0.8510 ± 0.0199	0.8129 ± 0.0326	0.8402 ± 0.0202	0.8176 ± 0.0205	0.8654 ± 0.0210
sPLS-DA	0.7703 ± 0.0347	0.8696 ± 0.0151	0.8327 ± 0.0239	0.8619 ± 0.0153	0.8360 ± 0.0142	0.8924 ± 0.0191
SVM-RFE	0.7776 ± 0.0209	0.8636 ± 0.0193	0.8471 ± 0.0169	0.8607 ± 0.0092	**0.8671 ± 0.0175**	0.8693 ± 0.0145

Values in bold represent the maximum value.

In summary, the validation results consistently indicate that the HCC DNBs identified by our method possess robust interpretability and can effectively differentiate the occurrence and development stages of HCC, thereby demonstrating the advancements and effectiveness of the proposed getDNB model.

## 4 Conclusions

In this article, we introduced a novel computational framework, getDNB, which utilizes a graph-embedding-based anomaly detection algorithm to identify DNBs for complex diseases from time-varying gene regulatory networks. In contrast to prior researches, our model emphasizes the comprehensive exploitation of characteristic information and structural alterations within dynamic networks. This approach has proven to be an efficient means of discovering HCC DNBs and provides substantial support for identifying diagnostic DNBs in other complex diseases. The source code and data can be found at https://github.com/zpliulab/getDNB.

By eliminating redundancy and linking the abnormal genes detected by our model, we successfully pinpointed 33 robust HCC DNBs and their interconnected networks. We validated the accuracy of our findings through various methods. Firstly, numerous identified HCC DNBs have been established as HCC-associated in existing literature. Secondly, functional enrichment analysis of these HCC DNBs underscored their significance in the onset and progression of HCC. More crucially, we introduced the concept of DNI and temporal classification techniques to further substantiate the validity and precision of the identified HCC DNBs within dynamic temporal networks. Furthermore, the robust performance of our model across multiple independent real-world datasets demonstrated its reliability. When compared to other feature selection as well as existing DNB discovery methods, our approach exhibited superior performance on several HCC datasets, confirming the efficiency and advantage of the proposed methods in identifying DNBs for disease progression.

In conclusion, getDNB stands as an effective approach for identifying DNBs from time-evolving dynamic networks. By adopting a fresh perspective, it fully capitalizes on variations in both network structure and information, demonstrating remarkable performance in the context of HCC during its occurrence and progression. In the future, we aim to explore more sophisticated graph embedding techniques and anomaly detection strategies to further refine the proposed getDNB model, enhancing its anomaly detection capabilities in complex disease DNBs. Additionally, we intend to broaden the application of the getDNB to other types of complex disease, as well as with single-cell sequencing data and spatial transcriptomics data.

## Supplementary Material

btaf518_Supplementary_Data
