# Supplementary material for: getDNB: identifying dynamic network biomarkers of hepatocellular carcinoma from time-varying gene regulations utilizing graph embedding techniques for anomaly detection
Source: Bioinformatics. 2025 Sep 15;41(9):btaf518. doi: 10.1093/bioinformatics/btaf518 (PMC12461858; doi:10.1093/bioinformatics/btaf518)
Supplement: btaf518_Supplementary_Data [file btaf518_supplementary_data.pdf]

getDNB: Identifying Dynamic Network Biomarkers of Hepatocellular Carcinoma from Time-Varying Gene Regulations Utilizing Graph Embedding Techniques for Anomaly Detection

Tong Wang[1] and Zhi-Ping Liu[1,*]

[1] *Department of Biomedical Engineering, School of Control Science and Engineering, Shandong University, Jinan, Shandong 250061, China.*

[*] *Correspondence: zpliu@sdu.edu.cn*

Supplementary Information

S1 Dataset details

Table S1 presents the detailed information of the HCC datasets used in this study, including the number of pathological samples, gene coverage, and classification of pathological stages. Based on the summarized characteristics of these datasets, we applied standardized preprocessing procedures to ensure comparability and facilitate integrative analysis across different platforms, namely Affymetrix microarray, Illumina microarray, and RNA-seq.

1. GSE6764 (Affymetrix Human Genome U133 Plus 2.0 Array): Raw CEL files were processed using the RMA (Robust Multi-array Average) algorithm through the affy R package. Probe IDs were mapped to gene symbols using the hgu133plus2.db annotation package. If multiple probes mapped to the same gene, the probe with the maximum expression value was retained. The final expression matrix was log2-transformed and exported for further analysis.

2. GSE89377 (Illumina HumanHT-12 V4.0 expression beadchip): Raw non-normalized data were processed using the limma package. Background correction and quantile normalization were performed with the neqc function. Expression values were then log2-transformed and normalized again across arrays using normalizeBetweenArrays to eliminate distribution differences.

3. TCGA-LIHC (RNA-Seq): Gene expression quantification data were downloaded using the TCGAbiolinks package. Gene-level count data were converted to gene symbols via Ensembl ID mapping using the bitr function from clusterProfiler. Redundant or unmatched genes were removed, ensuring one-to-one gene symbol mapping. The expression matrix was log2-transformed and exported for further analysis.

Table S1: Details of samples in the HCC datasets.

Datasets	AUPR	Remained genes	Number of used stages	Stages	Number in stages
GSE6764	72	20824	8	C	10
				Ci	10
				LDN	10
				HDN	7
				veHCC	8
				eHCC	10
				aHCC	7
				vaHCC	10
GSE89377	87	20805	7	C	13
				Ci	12
				LDN	11
				HDN	11
				eHCC	14
				aHCC	12
				vaHCC	14
TCGA-LIHC	396	36422	5	Stage 0	50
				Stage I	171
				Stage II	85
				Stage III	85
				Stage IV	5

S2 Details of crucial point extraction

Selecting anomaly genes based on a fixed proportion may introduce redundancy, and the resulting set of anomaly genes may not form a fully connected network. Therefore, the extraction of crucial points in the getDNB model is of great importance.

To address the redundancy issue within the anomaly gene set, we utilized the Depth-First Search (DFS) greedy algorithm to extract the minimum dominating set from the anomaly candidates. This greedy algorithm makes locally optimal choices at each step, aiming to achieve a globally optimal solution. In our model, the greedy strategy selects the vertex that covers the most uncovered vertices (i.e., the vertex with the highest degree or the most neighbors) in each iteration. The detailed steps of the algorithm are as follows.

Initialization: Create an empty dominant set S and a set U containing all uncovered nodes in the graph G (initially, all nodes V are uncovered),

$$S \leftarrow \emptyset,\ U \leftarrow V. \tag{1}$$

Iterative process: From the set U of uncovered nodes, select a node v that covers the most uncovered nodes (i.e., the node with the most neighbors), where $N(u)$ refers to all the neighbors of node u,

$$v \leftarrow \arg\max_{u \in U} |N(u) \cap U| . \tag{2}$$

Then, add node v to the dominant set S,

$$S \leftarrow S \cup \{v\}. \tag{3}$$

Update the uncovered node set U by removing node v and all its neighbors $N(u)$ from U,

$$U \leftarrow U - \{v\} - N(v). \tag{4}$$

Repeat the above steps until all nodes are covered. After completing the entire iteration process, we obtained a minimum dominating set of the anomaly gene set, which eliminates redundancy. To enhance clarity and reproducibility, the detailed pseudocode of the proposed algorithm is provided below.

Algorithm 1 The Minimum Dominating Set Algorithm Based on DFS

Input: Graph $G = (V, E)$
Output: Dominating set S
Initialize: $S \leftarrow \emptyset$, $U \leftarrow V$
while $U \neq \emptyset$ **do**
    for all $u \in U$ **do**
        Compute $coverage(u) \leftarrow |N(u) \cap U|$
    end for
    Select $v \leftarrow \arg\max_{u \in U} coverage(u)$
    $S \leftarrow S \cup \{v\}$
    $U \leftarrow U - \{v\} - N(v)$
end while
Return S

As the minimum dominating set may not inherently create a connected network, we utilize the shortest path algorithm to incorporate several anomaly gene nodes, ensuring the final DNBs constitute a connected network. In getDNB, we apply the Dijkstra algorithm to determine the shortest paths, with the specific process detailed below. Note that the Dijkstra algorithm has high time complexity, making it suitable for small-scale dominating sets.

Initialization: Input the graph G and specify the node set S (the minimum dominating set obtained from the previous step). Initialize an empty graph G' and an empty set N to store paths:

$$G' \leftarrow \emptyset, \ N \leftarrow \emptyset. \tag{5}$$

Iterative process: Through an iterative process, for each pair of nodes (s_i, s_j) in set S, find the shortest path $P(s_i, s_j)$ between them using the Dijkstra algorithm,

$$P(s_i, s_j) = shortest\_path(G, s_i, s_j). \tag{6}$$

Add all nodes on the shortest path to set N,

$$N = \cup_{s_i, s_j \in S} P(s_i, s_j). \tag{7}$$

Add all nodes from set S to graph G',

$$Nodes(G') = N. \tag{8}$$

In graph G, for each pair of nodes in set $N(u,v)$, if $(u,v) \in E$, add this edge to graph G',

$$Edges(G') = \{(u,v)|u,v \in N, (u,v) \in E\}. \tag{9}$$

After iterating through all node pairs in set S, we completed the crucial point extraction process and obtain the discovered HCC DNBs along with their interconnected network. To clearly illustrate the procedure used to ensure network connectivity, we presented the proposed algorithm of the shortest path based on Dijkstra below.

Algorithm 2 The Shortest Path Algorithm Based on Dijkstra

Input: Graph $G = (V, E)$, Dominating set S
Output: Connected subgraph $G' = (N, E')$
Initialize: $N \leftarrow \emptyset$, $G' \leftarrow$ empty graph
for all pairs $(s_i, s_j) \in S$ **do**
    Compute $P(s_i, s_j) \leftarrow$ shortest path in G using Dijkstra
    $N \leftarrow N \cup P(s_i, s_j)$
end for
for all $u, v \in N$ **do**
    if $(u,v) \in E$ **then**
        Add edge (u,v) to G'
    end if
end for
Return G'

S3 Supplementary details on other comparison methods

To evaluate the performance and robustness of our model, we conducted a comparative analysis against several widely-used feature selection methods, e.g., DUBStepR, RF-RFE, DEG (benchmark), sPLS-DA, SVM-RFE, and the existing DNB identification approaches such as IE-DNB and NIEE-DNB.

Specifically, DUBStepR, DEG, sPLS-DA, SVM-RFE, and RF-RFE are representative and commonly applied feature selection techniques in transcriptomics data analysis. In contrast, IE-DNB and NIEE-DNB are representative DNB discovery methods.

By including both categories—traditional feature selection techniques and advanced DNB-based models—our comparison ensures a fair and comprehensive evaluation. Below, we provided a brief description of each competing method to clarify their differences and relevance.

1. DUBStepR: An unsupervised method designed for RNA-seq data, selecting features that maximize cluster separability, which can realize feature selection and classification modeling simultaneously.

2. RF-RFE (Random Forest Recursive Feature Elimination): A tree-based wrapper method that selects features by iteratively removing the least important ones as ranked by a Random Forest classifier.

3. DEG (Differentially Expressed Genes): A traditional univariate statistical method that identifies features based on significant expression differences between groups.

4. sPLS-DA (sparse Partial Least Squares Discriminant Analysis): A multivariate model that simultaneously performs dimensionality reduction and feature selection.

5. SVM-RFE (Support Vector Machine Recursive Feature Elimination): A popular wrapper method using SVM to iteratively eliminate features with the lowest weights.

6. IE-DNB and NIEE-DNB: Both are dynamic network biomarker identification methods. They are direct graph-based methods that rely on statistical network measures.

For each method, we either adopted the default parameters recommended in the original publication or applied grid search-based tuning to ensure a fair performance evaluation. Table S2 details the hyperparameters selected for the compared methods.

Table S2: Hyperparameters used for comparison methods.

**Methods**	**Package**	**Parameter**	**Value**	**Description**
DUBStepR	DUBStepR	optimise.features	FALSE	Whether to optimize the number of selected features
RF-RFE	scikit-learn	n_estimators<br>random_state	100<br>42	The number of trees in the forest<br>Control randomness to ensure reproducible results
DEG(benchmark)	DESeq2	fitType<br>test	parametric<br>Wald	How to fit the dispersion model<br>Difference test type
sPLS-DA	mixOmics	ncomp<br>folds<br>nrepeat	10<br>5<br>10	Number of latent components<br>Number of folds in cross-validation<br>Number of repetitions for cross-validation to ensure stability
SVM-RFE	scikit-learn	kernel<br>random_state	linear<br>42	The Kernel type of SVM<br>Control randomness to ensure reproducible results
IE-DNB	/	maxclust	24	The number of hierarchical clusters is set to the same as the number of getDNB models in this article
NIEE-DNB	/	nlargest	100	The number of high-perturbation network edges selected in each sample

S4 Supplementary results

Table S3 summarizes the interpretation of 33 HCC DNBs identified by getDNB in existing literature related to HCC. Fig S1 depicts the expression levels of 25 HCC DNBs at temporal stages through violin plots, excluding the top 8 HCC DNBs with the highest degree that have been described in the main text. Fig S2 compares the overlapping genes in the gene sets selected by different feature selection methods and analyzed the KEGG and GO functional enrichments of biomarker gene sets selected by these comparing methods.

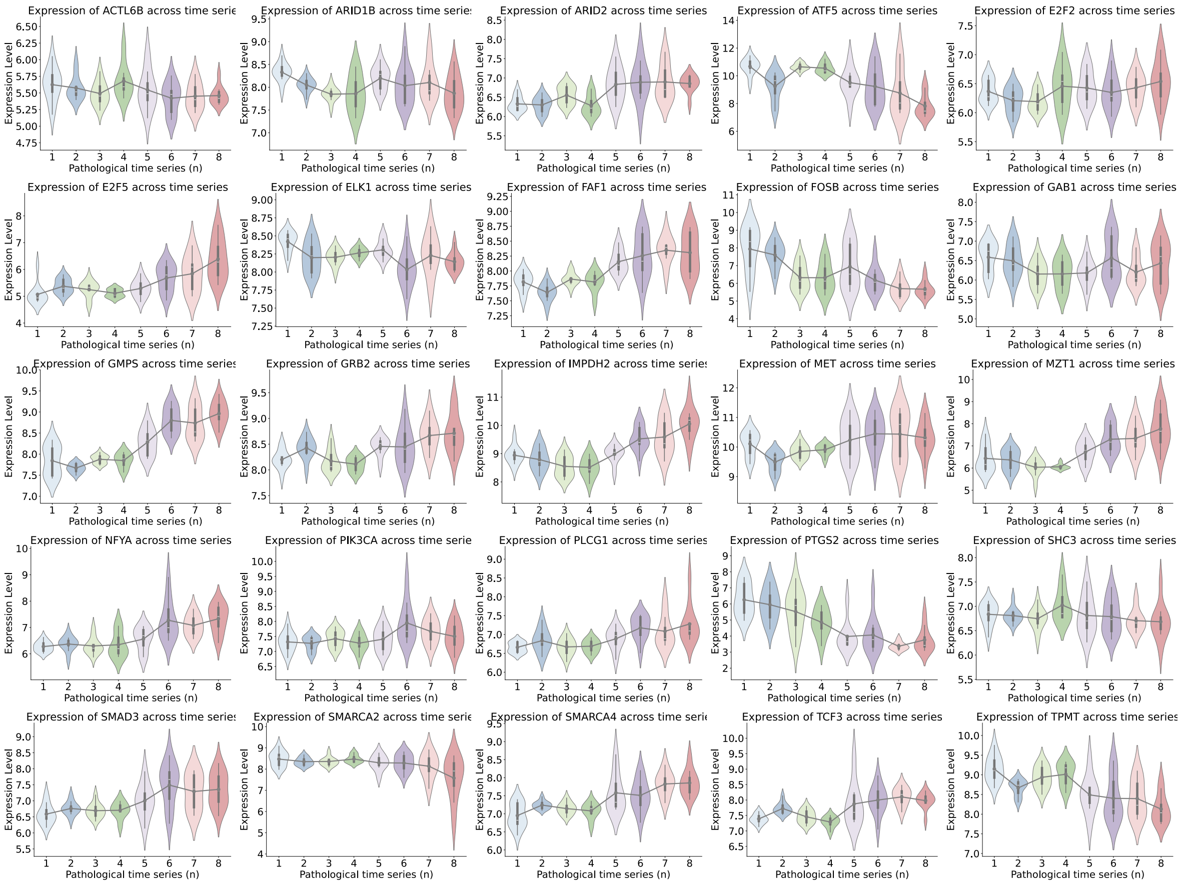

Figure S1: Violin plots depicted the expression levels of 33 HCC DNBs across different temporal stages, excluding the top 8 ones in degree ranking.

Table S3: Summary of the associations between the 33 HCC DNBs and HCC in existing literature.

No.	Gene Symbol	Gene ID	Gene Function	Involved	Detailed Research	DOI
1	ACTL6B	51412	Involved in transcriptional activation and repression of select genes by chromatin remodeling (alteration of DNA-nucleosome topology).	Y	Y	https://doi.org/10.1053/ j.gastro.2013.08.055
2	ARID1B	57492	Involved in transcriptional activation and repression of select genes by chromatin remodeling (alteration of DNA-nucleosome topology).	Y	Y	https://doi.org/10.18632 %2Foncotarget.16074
3	ARID2	196528	Involved in transcriptional activation and repression of select genes by chromatin remodeling (alteration of DNA-nucleosome topology)..	Y	Y	https://doi.org/10.18632 %2Foncotarget.355
4	ATF2	1386	Transcriptional activator which regulates the transcription of various genes, including those involved in anti-apoptosis, cell growth, and DNA damage response.	Y	Y	https://doi.org/10.1186 %2Fs12885-021-08540-x
5	ATF5	22809	Transcription factor that either stimulates or represses gene transcription through binding of different DNA regulatory elements.	Y	Y	https://doi.org/10.1158/ 0008-5472.CAN-07-6469

No.	Gene Symbol	Gene ID	Gene Function	Involved	Detailed Research	DOI
6	BRCA1	672	Plays a central role in DNA repair by facilitating cellular responses to DNA damage.	Y	Y	https://doi.org/10.1089/dna.2020.5644
7	CTNNB1	1499	Key downstream component of the canonical Wnt signaling pathway.	Y	Y	https://doi.org/10.1016/j.ygeno.2013.04.001
8	E2F2	1870	Transcriptional activators, found in the promoter region of many genes, whose products are involved in cell cycle regulation or DNA replication.	Y	Y	https://doi.org/10.1016/j.cellsig.2014.01.008
9	E2F4	1874	Transcriptional activators, found in the promoter region of many genes, whose products are involved in cell cycle regulation or DNA replication.	Y	Y	https://doi.org/10.1016/j.cellsig.2014.01.008
10	E2F5	1875	Transcriptional activator that binds to E2F sites, these sites are present in the promoter of many genes whose products are involved in cell proliferation.	Y	Y	https://doi.org/10.1016/j.cellsig.2014.01.008
11	EGR1	1958	Regulates the transcription of numerous target genes, and thereby plays an important role in regulating the response to growth factors, DNA damage, and ischemia. Plays a role in the regulation of cell survival, proliferation and cell death.	Y	Y	https://doi.org/10.3748/wjg.v8.i2.203

No.	Gene Symbol	Gene ID	Gene Function	Involved	Detailed Research	DOI
12	ELK1	2002	Transcription factor that binds to purine-rich DNA sequences.	Y	Y	https://doi.org/10.2147/OTT.S98727
13	FAF1	11124	Ubiquitin-binding protein.	N	N	/
14	FOS	2353	Nuclear phosphoprotein which forms a tight but non-covalently linked complex with the JUN/AP-1 transcription factor.	Y	Y	https://doi.org/10.1155/2020/6784138
15	FOSB	2354	Heterodimerizes with proteins of the JUN family to form an AP-1 transcription factor complex, thereby enhancing their DNA binding activity to gene promoters containing an AP-1 consensus sequence 5'-TGA[GC]TCA-3' and enhancing their transcriptional activity.	Y	Y	https://doi.org/10.21037%2Ftcr-23-829
16	GAB1	2549	Adapter protein that plays a role in intracellular signaling cascades triggered by activated receptor-type kinases.	Y	Y	hhttps://doi.org/10.1371/journal.pone.0085170
17	GMPS	8833	Catalyzes the conversion of xanthine monophosphate (XMP) to GMP in the presence of glutamine and ATP through an adenyl-XMP intermediate.	Y	Y	https://doi.org/10.1016/j.ajpath.2016.09.022

No.	Gene Symbol	Gene ID	Gene Function	Involved	Detailed Research	DOI
18	GRB2	2885	Adapter protein that provides a critical link between cell surface growth factor receptors and the Ras signaling pathway.	Y	Y	https://doi.org/10.1371/journal.pone.0085170
19	IMPDH2	3615	Catalyzes the conversion of inosine 5'-phosphate (IMP) to xanthosine 5'-phosphate (XMP), the first committed and rate-limiting step in the de novo synthesis of guanine nucleotides, and therefore plays an important role in the regulation of cell growth.	Y	Y	https://www.ncbi.nlm.nih.gov/pmc/articles/PMC6129487/
20	MET	4233	Receptor tyrosine kinase that transduces signals from the extracellular matrix into the cytoplasm by binding to hepatocyte growth factor/HGF ligand.	Y	Y	https://doi.org/10.1016/j.jhep.2013.09.009
21	MYC	4609	Transcription factor that binds DNA in a non-specific manner, yet also specifically recognizes the core sequence 5'-CAC[GA]TG-3'.	Y	Y	https://doi.org/10.1158/0008-5472.CAN-22-0232
22	MZT1	440145	Required for gamma-tubulin complex recruitment to the centrosome..	N	N	/

No.	Gene Symbol	Gene ID	Gene Function	Involved	Detailed Research	DOI
23	NFYA	4800	Component of the sequence-specific heterotrimeric transcription factor (NF-Y) which specifically recognizes a 5'-CCAAT-3' box motif found in the promoters of its target genes.	Y	Y	https://doi.org/10.3390/ijms21239157
24	PIK3R1	5295	Binds to activated (phosphorylated) protein-Tyr kinases, through its SH2 domain, and acts as an adapter, mediating the association of the p110 catalytic unit to the plasma membrane.	Y	Y	https://doi.org/10.1186/s40659-018-0202-7
25	PLCG1	5335	Mediates the production of the second messenger molecules diacylglycerol (DAG) and inositol 1,4,5-trisphosphate (IP3).	Y	Y	https://doi.org/10.1111/hepr.13309
26	PTGS2	5743	Dual cyclooxygenase and peroxidase in the biosynthesis pathway of prostanoids,with a particular role in the inflammatory response.	Y	Y	https://doi.org/10.1080/15548627.2019.1569934
27	SHC3	53358	Signaling adapter that couples activated growth factor receptors to signaling pathway in neurons. Involved in the signal transduction pathways of neurotrophin-activated Trk receptors in cortical neurons.	Y	Y	https://doi.org/10.1038%2Fs41419-021-03560-8

No.	Gene Symbol	Gene ID	Gene Function	Involved	Detailed Research	DOI
28	SMAD3	4088	Receptor-regulated SMAD (R-SMAD) that is an intracellular signal transducer and transcriptional modulator activated by TGF-beta (transforming growth factor) and activin type 1 receptor kinases.	Y	Y	https://doi.org/10.1016/j.toxlet.2014.08.024
29	SMARCA2	6595	Involved in transcriptional activation and repression of select genes by chromatin remodeling (alteration of DNA-nucleosome topology).	Y	Y	https://doi.org/10.1038%2Fs41598-018-20217-3
30	SMARCA4	6597	Involved in transcriptional activation and repression of select genes by chromatin remodeling (alteration of DNA-nucleosome topology).	Y	Y	https://doi.org/10.1038%2Fs41598-018-20217-3
31	TCF3	6929	Transcriptional regulator involved in the initiation of neuronal differentiation and mesenchymal to epithelial transition.	Y	Y	https://doi.org/10.1016/j.hbpd.2022.01.003
32	TP53	7157	Acts as a tumor suppressor in many tumor types; induces growth arrest or apoptosis depending on the physiological circumstances and cell type.	Y	Y	https://doi.org/10.1016/j.ygeno.2013.04.001
33	TPMT	7172	Catalyzes the S-methylation of thiopurine drugs such as 6-mercaptopurine and 6-thioguanineusing S-adenosyl-L-methionine as the methyl donor.	N	N	/

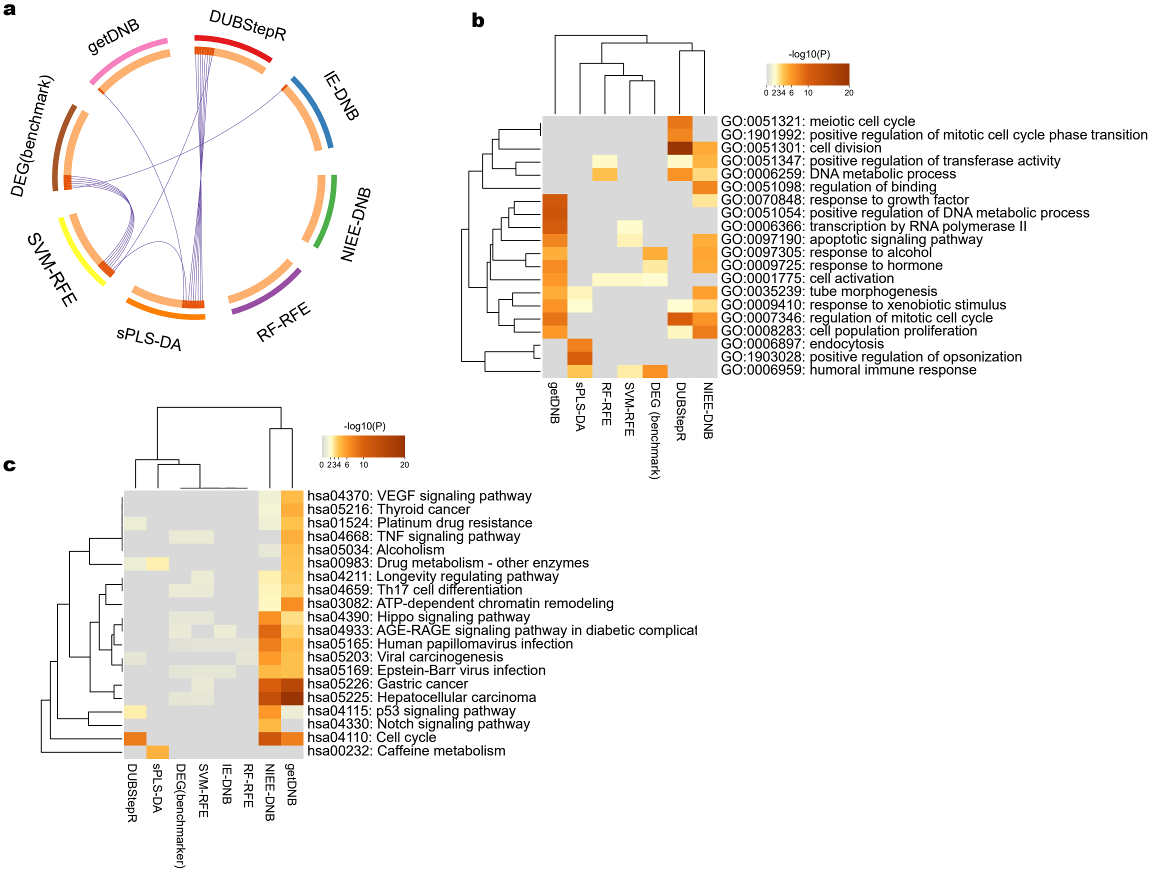

Figure S2: **Comparison of feature selection methods.** (a) Venn diagram showing the overlap of gene sets selected by various feature selection methods. (b) KEGG pathway enrichment analysis of the top 20 pathways for gene subsets selected by the six methods. (c) GO functional enrichment analysis of the top 20 functional terms for the same gene subsets. Gray cells in (b) and (c) indicate non-significant enrichment results.
